# Factors Associated with Home Fire Escape Plans in New South Wales: Multinomial Analysis of High-Risk Individuals and New South Wales Population

**DOI:** 10.3390/ijerph15112353

**Published:** 2018-10-25

**Authors:** W. Kathy Tannous, Kingsley E. Agho

**Affiliations:** 1 School of Business, Western Sydney University, Parramatta, NSW 2150, Australia; 2 School of Science and Health, Western Sydney University, Locked Bag 1797, Penrith, NSW 2571, Australia; k.agho@westernsydney.edu.au

**Keywords:** home fire, escape plan, fire safety, high-risk individual, New South Wales

## Abstract

The preparation and practice of home-escape plans are important strategies for individuals and families seeking to reduce and/or prevent fire-related injury or death. The aim of this study was to assess the prevalence of and factors associated with, home-escape plans in the state of New South Wales (NSW), Australia. The study used data from two surveys—a 2016 fire safety attitudes and behaviour survey administered to high-risk individuals (*n* = 296) and a 2013 NSW health survey covering 13,027 adults aged 16 years and above. It applied multinomial logistic regression analyses to these data to identify factors associated with having a written home-fire escape plan, having an unwritten home-fire escape plan and not having any home-fire escape plan. The prevalence of written home-escape plans was only 4.3% (95% CI: 2.5, 7.5) for the high-risk individuals and 7.9% (95% confidence interval [CI]: 7.3, 8.6) for the entire NSW population. The prevalence of unwritten escape plans was 44.6% (95% CI: 38.8, 50.5) for the high-risk individuals and 26.2% (95% CI: 25.1, 27.2) for the NSW population. The prevalence of no-escape plan at all was 51.1% (95% CI: 45.2, 56.9) for the high-risk individuals and 65.9% (95% CI: 64.8, 67.1) for the NSW population. After adjusting for other covariates, the following factors were found to be significantly associated with unwritten-escape plan and no-escape plan prevalence: speaking only the English language at home, practicing home-fire escape plans infrequently, being married, being female and testing smoke alarms less often. Future fire interventions should target people who speak only English at home and people who test their smoke alarms infrequently. These interventions should be accompanied by research aimed at reversing the trend toward use of more flammable materials in homes.

## 1. Introduction

In financial year 2016–2017 (FY17), fire-fighting agencies in Australia attended 95,508 fire events. Of these, 17,043 events (18% of the total) related to structure fires (fires that occur inside a building or other structure, regardless of whether there is damage to the structure [1]. In NSW, Australia’s largest state, the number of fire events was 33,856 of which 6414 were structure fires (19%) and 6013 were responded to by Fire & Rescue NSW (FRNSW) [1]. For structure fires in NSW, the time from receipt of phone call by the fire-fighting agency to arrival of the first fire crew at the fire scene averaged 7.5 min, with response time being slightly lower in the major cities and higher in regional and remote areas [1].

The costs of fire are multi-fold, ranging from property damage to loss of life, physical injury and mental trauma. In 2017, insurance claims for fire damage to residential properties amounted to $176 million in NSW and $565 million in Australia as a whole. In NSW, the number of fire-related deaths was 24 (3.1 per million persons) and of hospital admissions due to fire-related injuries was 1005 (131 per million persons) [1]. In addition, both physical and mental costs were incurred by first responders from the fire brigades, police forces and paramedic services. The recurring exposure of firefighters to physically-painful and emotionally-disturbing experiences has been shown to contribute to posttraumatic stress symptoms (PTSS) and to aggravate their associated risks [2].

A new and disturbing factor that needs to be considered by households preparing themselves for a possible residential fire emergency is the greatly reduced escape time that has resulted from changes in furnishings, housing styles and security measures [3]. In recent tests of fires using piloted-flaming combustion, the average time taken for temperatures to reach 65 °C at ceiling height was 130 s (two minutes and 10 s). In similar tests undertaken in 1975, the corresponding time was 970 s (16 min and 10 s) [3]. The faster development of fires is due mainly to the use of more flammable materials in home furniture, such as upholstered couches and mattresses [3,4]. In countries, like the United Kingdom, they have the requirement of treating home furniture with flame retardants. However, in a recent study by McKenna et al. (2018), they determine that once fire ignition occurs then flame retardants has little effect on the fire growth rate [5].

Many residential fires and the injuries that ensue from them may be preventable by the use of early warning systems. An effective, reliable and cheap way to provide an early warning about home fires is to install and regularly check a smoke alarm [6]. The official recommendation is that smoke alarms be tested every month and that batteries in conventional smoke alarms be replaced every year [7,8].

Fires can grow and spread rapidly through a house. It is therefore crucial that residents are well-prepared to react quickly and appropriately to the activation of a smoke alarm [9]. Prevention strategies in fire-safety education programs include developing a home-fire escape plan [10]. The plan needs to be rehearsed frequently [11,12,13,14]. Studies in the United States [8,15] have recommended that home-fire escape plans be practiced at least twice a year. According to those studies, the plan should contain at least two different ways of escape for each resident of a household, as well as designation of a safe meeting place outside the residence after escaping the fire. The plan should ensure that every family member is capable of opening doors and windows to escape. It should also reaffirm the importance of family members reacting appropriately to the sound of an activated smoke alarm and provide direction for children and older persons on how to escape a burning home on their own. Studies on persons’ reaction to the odour and/or the sound of fire alarm alerting them to the possibility of fire risk conclude that persons would favour a delayed evacuation. This has been classified in the following categories of vulnerability: factors of the persons in terms of immobility or frailty, level of knowledge or training, aggravating factors of falling asleep or being under the influence of alcohol or drugs or impacted by medication uses [10,16].

There is only a small number of studies in the extant literature about home-fire escape plans. Most of these studies have been conducted in the United States [9,17]. In Australia, there have not been any studies of home-fire escape plans. The current paper partly redresses this paucity by assessing the prevalence of and factors associated with, home-fire escape plans in NSW for two populations, namely, high-risk individuals and the entire New South Wales populace. The study is intended to provide guidance to policy makers, particularly the Fire Rescue of New South Wales (FRNSW), on how to educate individuals in the implementation of safety procedures and reduce complacency among those who aware of their risks.

## 2. Methods

### 2.1. Data Source

The NSW Population Health Survey is a cross-sectional, computer-assisted telephone survey stratified by geographical regions. The target population is all residents of NSW through the use of overlapping dual-frame design with three types of phone use: landline only, mobile only and both mobile and landline in the same house. Participants were selected through either the landline or mobile phone number sampling frames [18]. The Home Fire Resilience Project (HFRP) targeted the most vulnerable and isolated members of the community. The program was focused on highly isolated (geographical and/or socially) older people and on average had some mobility difficulties. The physical or cognitive impairment as well as social isolation were detailed as risk factors for individuals or creation of vulnerability by Halvorsen et al. (2017) [19]. These are detailed in this paper as high-risk individuals and were selected for the project as they lived by themselves [20]. This population group had the risk factors that were identified by Turner et al. (2016) associated with unintentional house fires [21]. The NSW Population Health dataset consisted of 13,323 survey respondents while the HFRP dataset involved 296 survey respondents.

### 2.2. Conceptual Framework and Study Variables

**Figure 1:**
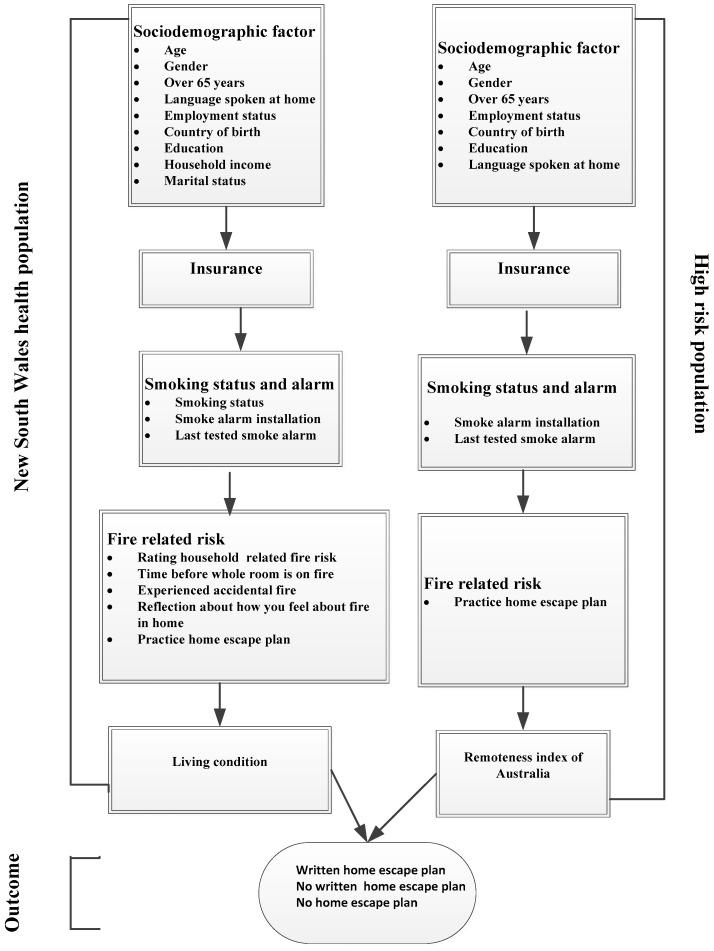
Conceptual model framework.

A contextual framework was adapted to group the factors potentially associated with having a written home-fire escape plan, an undocumented home-fire escape plan and no home-fire escape plan (Figure 1). Twenty potential risk factors were identified and divided into four categories: socio-demographic, insurance, smoking and smoke-alarm factors (Figure 1).

## 3. Statistical Analysis

Frequency tabulations were made to describe the data sets used in the study. The prevalence tables were then analysed to identify the impact of potential predictors on home-fire escape plans. Univariate and multivariate multinomial logistic regressions were used for multivariable analyses to ascertain associated outcome factors after adjusting for potential confounding variables. As part of the multivariable analysis, a five-stage model was developed along the lines of the conceptual model described in Figure 1. In the first stage of the model, socio-demographic factors were entered to assess their associations with the study outcomes. A manually-executed, backward-elimination method was used to identify factors significantly associated with the outcomes. In the second stage of the model, insurance was added to the significant factors in the first stage and this was followed by a backward-elimination procedure. A similar approach was used in later stages of the model for the smoking, smoke alarm and living/remoteness factors. The odds ratios with 95% CIs were calculated in order to assess the adjusted odds of independent variables and only those independent variables with *p* < 0.05 were retained in the final model. All analyses were conducted using STATA version 14.1 (STATA Corporation, College Station, TX, USA).

## 4. Results

### 4.1. Characteristics of the Respondents

The demographics of the respondents in the two studies are presented in Table 1. The majority were females, for both the high-risk individuals and the entire NSW population categories. Most respondents spoke only English in the home. Approximately 61% of the high-risk individuals were aged over 65 years compared with 32% for the NSW population. In both categories, more than 70% of respondents were born in Australia. More than half were insured and had carried out their last smoke-alarm test within six months of the surveys. Persons currently smoking were a minority among the NSW population. More than two-thirds of respondents in each survey category used battery-powered smoke alarms and more than three-quarters had practiced a home-fire escape plan in the last year. Most high-risk individuals owned their homes, had never experienced an accidental home fire and had low risk to fire hazard. However, they lived in remote or very remote parts of Australia and rooms in their houses would be fully engulfed in flames within five minutes of the fire starting.

**Figure 2:**
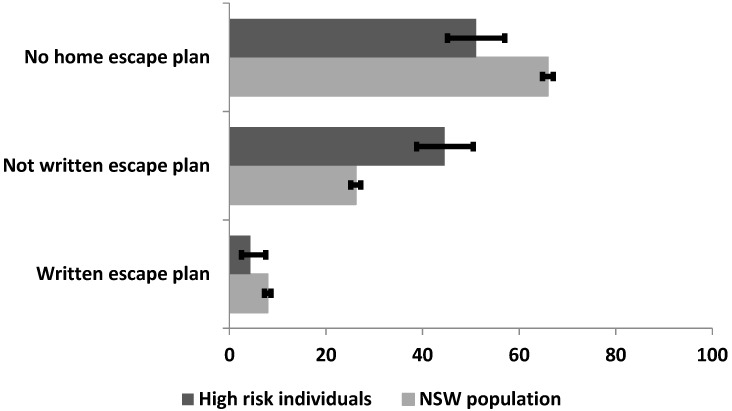
Prevalence of not possession of a home escape plan, not having a written home escape plan and having a home escape plan in NSW.

Figure 2 shows the prevalence of the three types of home-escape plan (written plan, unwritten plan and no plan) among the high-risk individuals and the NSW population. For both population groups, prevalence was highest for the category that had no escape plan at all and lowest for the category that had a conceptual plan they had put in writing.

### 4.2. Prevalence of Home-Fire Escape Plans

Table 2 presents the prevalence of home-fire escape plans among both high-risk individuals and the entire NSW population. Among the high-risk individuals, the prevalence of having a written fire-escape plan was significantly similar for those who had thought a lot and those who had thought little about fire risk. However, the prevalence of having an unwritten plan was significantly higher among those who had thought a lot about fire risk than among those who had thought little about such risk. In addition, the prevalence of both having a written plan and an unwritten plan was significantly higher for those who had practiced their plan within the last year compared to those who had not practiced a plan for one year or more. The prevalence of having no escape plan was significantly higher for females than males. 

For the whole NSW population, the prevalence of having a written escape plan was slightly higher, while that of having an unwritten plan was significantly lower, for males compared to females; the prevalence of not having any escape plan was therefore significantly higher for males. The prevalence of having no escape plan was significantly higher for those who were uninsured compared to those who were insured, whilst the prevalence of having an unwritten escape plan was significantly higher for those who were insured. The prevalence of having a written or unwritten escape plan was significantly higher for respondents who last tested their smoke alarm less than six months prior to the survey compared to those who had not tested their alarm during that period. Also, the prevalence of having a written escape plan was significantly higher for those who had practiced their escape plan less than a year prior to the survey compared to those who only practiced it one year or more prior to the survey.

### 4.3. Unadjusted Estimates of Home-Fire Escape Plans

Table 3 shows the unadjusted relative risks for factors associated with home-fire escape plans among both the high-risk individuals and the NSW population. For the high-risk individuals, speaking only English at home, being born in Australia and not having thought much about the possibility of a home-fire were significant risk factors for not having a written home-fire escape plan.

For the NSW population, being female, born in Australia, speaking only English at home, married, insured, earning more than $80,000 a year and not testing smoke alarms frequently were significant risk factors for not having a written home-fire escape plan.

### 4.4. Factors Associated with Home-Fire Escape Plans

Among the high-risk individuals, the likelihood of not having a written home-fire escape plan was significantly lower for those speaking both English and other languages at home compared to those speaking only English at home. For this category of respondents, the risk of not having a home-fire escape plan was significantly higher for those who had not practiced a fire escape plan within the last year.

Among the NSW population, the risk of having an unwritten fire-escape plan was significantly lower for those speaking English and other languages at home compared to those who spoke only English at home (Table 4). Married people had a significantly higher risk of not having a written home-fire escape plan compared to never-married people. The risk of not having a written home-fire escape plan and not having any home-fire escape plan was significantly higher among respondents who used batteries in their smoke alarms compared to those who used hardwired systems. Furthermore, there was a significantly lower risk of not having a written home-fire escape plan and not having any home-fire escape plan among people who tested their smoke alarms more frequently compared to those who did not. 

## 5. Discussion

This study used two separate surveys to assess the factors associated with home-fire escape plans, using multinomial regression analysis. The main risk factors associated with home-fire escape plans included: use of English language only at home, less regular practice of home-fire escape plans, being married and lower frequency of testing home-fire alarms.

Past research has shown that language spoken at home is generally not associated with health and safety measures [22]. However, in a contrary study in the United States [23], it was found that, compared to those who speak English at home, people who usually speak another language or who speak English and another language are at greater risk of having health problems or being susceptible to other factors. In this study we found that, for both the high-risk individuals and the NSW population, the hazard of having a home-fire escape plan but not writing it down and not having any home-fire escape plan was significantly higher for those whose spoke only English at home compared to those who spoke English and other languages at home. Those who spoke only English at home are most likely to be non-migrants who may have become complacent to such an extent that they would not fully appreciate the risks in not having a formal home-fire escape plan.

Married couples have been found to be much more likely to use child safety seats than people who are not married [24]. This finding buttresses the view that married people are more concerned about safety than their never-married counterparts. Studies have suggested that marriage may reduce risk-taking behaviour and accident risk by strengthening social bonds [25,26]. Contrary to these findings, our study found that married people were at a significantly higher danger of not having a written or unwritten home-fire escape plan compared to those who had never been married. The reason for this finding is not apparent and further research will be required to understand it fully.

Hardwired smoke detectors are thought to be more effective than battery-powered detectors; they are more dependable because the battery-powered alarm will eventually run out of power [27]. Residents who use battery-powered alarms may generally be less concerned about safety than those who use hardwired alarms. This is demonstrated in our study, which showed that people who used battery-powered alarms had a significantly higher risk of not having a written home-fire escape plan and not having a home-fire escape plan at all compared to those who used hardwired alarms.

In addition, we found that respondents who tested their smoke alarms less frequently were at a significantly higher risk of not having a written or unwritten home-fire escape plan compared to those who tested their alarms more frequently. This finding could be attributed to the fact that people who test their smoke alarms more frequently are more safety-oriented and would therefore be more likely to have a home-fire escape plan.

A key strength of our study of the NSW population was that it had a sample size large enough to permit generalisations covering the whole state. In contrast, the study of high-risk individuals had a small sample size, preventing generalisation of the results to all high-risk individuals in NSW.

## 6. Conclusions

In conclusion, this study found that the danger of not having a written home-fire escape plan was significantly higher among people who spoke only English at home. Other characteristics significantly associated with not having a written home-fire escape plan included: being married, using battery-powered smoke alarms and frequent testing of smoke alarms.

Future fire interventions should consider education programs for single females and married couples that explain the benefits and methods of reducing risk-taking behaviours towards home-fire hazards. Residents who use smoke alarms should be encouraged to make fire-escape plans and to document these plans on paper or in their computer. Further, people who test their smoke alarms more frequently should be reminded that it is still important to have a home-fire escape plan, to document the plans and to practice it with all members of the household.

There may also be a case for encouraging research into the development of materials that reverse the recent trend toward fire-promoting structures and furnishings. Progress with nanotechnology is leading to the discovery of “smart” materials with novel characteristics. It may be possible to develop fire-retardant materials that could form part of structures and furnishings or be coatings to these, giving home-occupants more time to escape burning buildings. It might also be possible to accelerate the development of smart materials that prevent door locks from expanding rapidly and preventing occupants escaping through doors.

## Figures and Tables

**Table 1:** Characteristics of the study respondents.

Variables	High-Risk Individuals *n* = 296	NSW Population *n* = 13,027
Demographic	*n* (%)	*n* (%)
Gender		
Male	95 (33.1)	5416 (41.6)
Female	192 (66.9)	7611 (58.4)
Age	57.1 (16.6)	54.0 (18.3)
Language spoken at home		
English	241 (83.1)	10,741 (85.9)
English plus other languages	49 (16.9)	1765 (14.1)
Over 65 years		
No	116 (39.2)	8841 (67.9)
Yes	180 (60.8)	4186 (32.1)
Current employment status		
Employed	12 (43.1)	6987 (53.9)
Not employed	161 (56.9)	5969 (46.1)
Country of birth		
Australia	230 (79.6)	9204 (70.6)
Others	59 (20.4)	3823 (29.4)
Education status		
Uni	68 (24.4)	3999 (31.1)
TAFE	59 (21.1)	3512 (37.3)
SC	152 (54.5)	5347 (41.6)
Household income (before tax)		
<$20,000		2076 (20.5)
$20,000–40,000		2139 (21.2)
$40,000–60,000		1382 (13.7)
$60,000–80,000		1161 (11.4)
>$80,000		3340 (33.0)
Marital status		
Married		6711 (51.9)
Widowed		1473 (11.4)
Separated		1911 (14.8)
Never married		2840 (21.9)
Insurance		
No	132 (44.6)	5486 (42.5)
Yes	164 (55.4)	7423 (57.5)
Smoking status		
Never		6293 (48.4)
Ever		4882 (37.5)
Now		1826 (14.1)
Smoke Alarm		
Smoke alarm installation		
Battery	191 (66.3)	8137 (68.1)
Hardwire	51 (17.7)	2347 (19.7)
Both	46 (16)	1463 (12.2)
Last tested smoke alarm		
Less than 6 months	64 (21.6)	7802 (59.9)
6 months or over	232 (78.4)	5225 (40.1)
Attitude towards fires		
Rating household related fire risk		
Low-risk	160 (54.1)	
High-risk	136 (45.9)	
Fire awareness		
Time have before whole room is on fire		
<5 min	186 (72.1)	
5–15 min	72 (27.9)	
Experienced unintentional or accidental fire		
Yes	40 (13.9)	
No	247 (86.1)	
Reflection about how you feel about fire in home		
High thought risk	195 (65.9)	
Low thought risk	101 (34.1)	
Practice home escape plan		
<1 year	54 (24.4)	1169 (9.0)
1 year or more	167 (75.6)	11,760 (91.0)
Living condition		
Own	213 (74)	
Rent	75 (26)	
Remoteness Index of Australia		
Highly accessible		1969 (15.1)
Accessible		2224 (17.1)
Moderately accessible		2800 (21.5)
Remote/Very remote		6028 (46.3)

**Table 2:** Prevalence of home escape plan according to characteristics of the respondents.

Variables	High-Risk Individuals				New South Wales (NSW) Population			
	Does Your Household Have Home Escape Plan				Does Your Household Have Home Escape Plan			
	Written	Not Written	No Home Escape Plan	*p*-Value	Written	Not Written	No Home Escape Plan	*p*-Value
	% [95% CI]	% [95% CI]	% [95% CI]		% [95% CI]	% [95% CI]	% [95% CI]	
Gender								
Male	2.3 [0.6, 8.8]	47.1 [36.8, 57.7]	50.6 [40.1, 61.0]		7.9 [7.2, 8.6]	24.0 [22.9, 25.2]	68.1 [66.9, 69.4]	
Female	5.5 [3.0, 10.0]	41.4 [34.4, 48.8]	53.0 [45.7, 60.2]	0.394	7.6 [7.0, 8.2]	28.6 [27.6, 29.6]	63.8 [62.7, 64.9]	<0.001
Age in years (mean, sd)	59.8 (15.4)	58.4 (14.6)	54.3 (17.5)	0.096	53.1 (20.4)	55.1 (16.9)	53.7 (18.5)	<0.001
Language spoken at home								
English	3.5 [1.8, 6.9]	46.5 [40.0, 53.0]	50.0 [43.5, 56.5]		7.4 [6.9, 7.9]	28.6 [27.7, 29.5]	64.0 [63.1, 64.9]	
English plus other languages	8.9 [3.4, 21.5]	31.1 [19.3, 46.0]	60.0 [45.1, 73.2]	0.077	10.5 [9.1, 12.0]	18.9 [17.1, 20.9]	70.6 [68.4, 72.8]	<0.001
Over 65 years								
No	5.8 [2.6, 12.4]	48.5 [39.0, 58.2]	45.6 [36.2, 55.4]		7.5 [7.0, 8.1]	26.2 [25.3, 27.1]	66.3 [65.3, 67.2]	
Yes	3.5 [1.6, 7.5]	42.2 [35.0, 49.7]	54.3 [46.8, 61.7]	0.308	8.1 [7.3, 9.0]	27.7 [26.3, 29.1]	64.3 [62.8, 65.7]	0.0834
Current employment status								
Employed	2.5 [0.8, 7.6]	42.4 [33.7, 51.5]	55.1 [46.0, 63.9]		7.4 [6.8, 8.0]	27.3 [26.2, 28.4]	65.3 [64.2, 66.4]	
Not employed	6.1 [3.2, 11.4]	45.6 [37.7, 53.7]	48.3 [40.3, 56.4]	0.276	8.1 [7.4, 8.8]	26.1 [24.9, 27.2]	65.9 [64.6, 67.1]	0.1433
Country of birth								
Australia	3.2 [1.5, 6.6]	47.0 [40.4, 53.7]	49.8 [43.1, 56.4]		7.2 [6.7, 7.8]	28.7 [27.8, 29.6]	64.1 [63.1, 65.1]	
Others	9.4 [4.0, 20.8]	32.1 [20.9, 45.8]	58.5 [44.8, 70.9]	0.041	8.9 [8.0, 9.9]	21.7 [20.4, 23.1]	69.4 [67.9, 70.9]	<0.001
Educational status								
Uni	2.9 [0.7, 11.1]	41.2 [30.1, 53.2]	55.9 [43.9, 67.2]		7.7 [6.9, 8.6]	28.3 [26.9, 29.7]	64.0 [62.5, 65.5]	
TAFE	5.3 [1.7, 15.2]	52.6 [39.7, 65.2]	42.1 [30.0, 55.2]		7.4 [6.5, 8.3]	30.0 [28.5, 31.5]	62.7 [61.0, 64.3]	<0.001
SC	3.6 [1.5, 8.3]	42.1 [34.2, 50.5]	54.3 [45.9, 62.4]	0.547	7.9 [7.2, 8.6]	23.6 [22.5, 24.8]	68.5 [67.2, 69.7]	
Household income (before tax)								
<$20,000					8.7 [7.5, 10.0]	24.7 [22.8, 26.6]	66.7 [64.6, 68.7]	
$20,000–40,000					7.3 [6.2, 8.5]	28.9 [27.0, 30.9]	63.8 [61.8, 65.9]	
$40,000–60,000					7.9 [6.6, 9.5]	26.8 [24.5, 29.2]	65.3 [62.7, 67.8]	0.0131
$60,000–80,000					6.8 [5.5, 8.5]	27.6 [25.1, 30.3]	65.5 [62.7, 68.2]	
>$80,000					6.8 [6.0, 7.7]	29.1 [27.6, 30.7]	64.1 [62.5, 65.8]	
Marital status								
Married					7.0 [6.4, 7.6]	29.1 [28.0, 30.2]	64.0 [62.8, 65.1]	
Widowed					9.3 [7.9, 11.0]	24.6 [22.4, 26.9]	66.1 [63.6, 68.5]	
Separated					6.9 [5.8, 8.1]	27.5 [25.5, 29.5]	65.7 [63.5, 67.8]	
Never married					9.3 [8.3, 10.5]	21.5 [20.0, 23.1]	69.2 [67.4, 70.9]	
Insurance								
No	4.2 [1.7, 9.7]	40.8 [32.4, 49.9]	55.0 [46.0, 63.7]		8.0 [7.3, 8.8]	24.5 [23.3, 25.6]	67.5 [66.2, 68.7]	
Yes	4.5 [2.1, 9.2]	47.4 [39.7, 55.3]	48.1 [40.3, 55.9]	0.520	7.4 [6.8, 8.0]	28.4 [27.3, 29.4]	64.3 [63.2, 65.4]	<0.001
Smoking status								
Never					7.9 [7.3, 8.6]	26.7 [25.6, 27.9]	65.3 [64.1, 66.5]	
Ever					7.2 [6.5, 8.0]	27.7 [26.4, 29.0]	65.1 [63.8, 66.5]	0.0169
Now					8.2 [7.0, 9.6]	23.7 [21.8, 25.8]	68.0 [65.8, 70.2]	
Smoke Alarm								
Smoke alarm installation								
Battery	5.1 [2.7, 9.6]	41.7 [34.6, 49.2]	53.1 [45.7, 60.5]		6.9 [6.4, 7.5]	26.0 [25.1, 27.0]	67.1 [66.0, 68.1]	
Hardwire	0	59.2 [45.0, 72.0]	40.8 [28.0, 55.0]	0.178	9.9 [8.8, 11.2]	28.6 [26.8, 30.5]	61.4 [59.4, 63.4]	<0.001
Both	4.3 [1.1, 15.9]	43.5 [30.0, 58.0]	52.2 [37.9, 66.1]		9.3 [7.9, 10.9]	34.5 [32.1, 37.0]	56.1 [53.6, 58.7]	
Last tested smoke alarm								
Less than 6 months	7.9 [3.3, 17.8]	50.8 [38.6, 62.9]	41.3 [29.8, 53.8]		8.9 [8.2, 9.5]	29.9 [28.8, 30.9]	61.3 [60.2, 62.4]	
6 months or over	3.3 [1.6, 6.8]	42.7 [36.2, 49.5]	54.0 [47.2, 60.6]	0.099	6.0 [5.3, 6.6]	21.8 [20.7, 23.0]	72.2 [71.0, 73.4]	<0.001
Attitude towards fires								
Rating household related fire risk								
Low-risk	3.4 [1.4, 8.1]	42.8 [34.9, 51.0]	53.8 [45.6, 61.8]					
High-risk	5.3 [2.6, 10.8]	46.6 [38.2, 55.2]	48.1 [39.6, 56.7]	0.542				
Fire awareness								
Time have before whole room is on fire								
<5 min	4.5 [2.2, 8.7]	42.5 [35.4, 49.9]	53.1 [45.7, 60.3]					
5–15 min	4.3 [1.4, 12.7]	53.6 [41.8, 65.1]	42.0 [30.9, 54.0]	0.278				
Experienced unintentional or accidental fire								
Yes	2.6 [0.4, 16.3]	41.0 [26.8, 56.9]	56.4 [40.6, 71.0]					
No	3.8 [2.0, 7.3]	45.3 [39.0, 51.8]	50.9 [44.4, 57.3]	0.785				
Reflection about how you feel about fire in home								
High thought risk	4.3 [2.2, 8.5]	51.6 [44.4, 58.8]	44.0 [37.0, 51.3]					
Low thought risk	4.3 [1.6, 11.1]	30.4 [21.9, 40.6]	65.2 [54.9, 74.3]	0.003				
Practice home escape plan								
<1 year	15.7 [8.0, 28.5]	78.4 [65.0, 87.7]	5.9 [1.9, 16.8]		36.0 [33.3, 38.8]	64.0 [61.2, 66.7]	0	
1 year or more	2.5 [0.9, 6.6]	46.5 [38.9, 54.4]	50.9 [43.1, 58.7]	<0.001	16.9 [15.6, 18.3]	83.1 [81.7, 84.4]	0	<0.001
Living condition								
Own	4.9 [2.6, 8.8]	45.1 [38.4, 52.0]	50.0 [43.2, 56.8]					
Rent	3.1 [0.8, 11.6]	38.5 [27.4, 50.8]	58.5 [46.1, 69.8]	0.465				
Remoteness Index of Australia								
Highly accessible					7.3 [6.2, 8.6]	26.2 [24.3, 28.2]	66.5 [64.3, 68.6]	
Accessible					7.5 [6.4, 8.6]	26.7 [24.9, 28.6]	65.8 [63.8, 67.8]	
Moderately accessible					8.2 [7.2, 9.3]	28.0 [26.3, 29.7]	63.8 [62.0, 65.6]	0.4613
Remote/Very remote					7.7 [7.1, 8.4]	26.2 [25.1, 27.3]	66.1 [64.9, 67.3]	

**Table 3:** Home fire escape plan. Univariate analysis.

Variables	High-Risk Individuals								New South Wales (NSW) Population							
	Not Written				No Home Escape Plan				Not Written				No Home Escape Plan			
	OR	95% CI		*p*-Value	OR	95% CI		*p*-Value	OR	95% CI		*p*-Value	OR	95% CI		*p*-Value
Gender																
Male	1.00				1.00				1.00				1.00			
Female	0.37	0.08	1.75	0.208	0.44	0.09	2.08	0.297	1.19	1.04	1.36	0.010	0.98	0.86	1.11	0.740
Age (Continuous)	0.99	0.96	1.03	0.785	0.98	0.94	1.02	0.270	1.00	1.00	1.00	0.980	1.00	1.00	1.01	0.035
Language spoken at home																
English	1.00				1.00				1.00				1.00			
English plus other languages	0.27	0.07	0.99	0.050	0.48	0.13	1.70	0.255	0.49	0.40	0.59	<0.001	0.84	0.71	0.99	0.040
Over 65 years																
No	1.00				1.00				1.00				1.00			
Yes	1.46	0.45	4.79	0.532	2.00	0.61	6.54	0.251	0.92	0.80	1.07	0.277	0.93	0.82	1.07	0.323
Current employment status																
Employed	1.00				1.00				1.00				1.00			
Not employed	0.45	0.11	1.74	0.244	0.36	0.09	1.40	0.142	0.87	0.76	1.01	0.062	0.92	0.81	1.05	0.227
Country of birth																
Australia	1.00				1.00				1.00				1.00			
Others	0.23	0.07	0.82	0.023	0.40	0.12	1.35	0.141	0.60	0.52	0.69	<0.001	0.92	0.80	1.05	0.199
Educational status																
Uni	1.00				1.00				1.00				1.00			
TAFE	0.71	0.11	4.60	0.723	0.42	0.07	2.71	0.362	1.11	0.92	1.34	0.283	1.03	0.86	1.22	0.784
SC	0.84	0.15	4.62	0.844	0.80	0.15	4.32	0.795	0.82	0.69	0.97	0.021	1.05	0.90	1.23	0.546
Household income (before tax)																
<$20,000									1.00					1.00		
$20,000–40,000									1.39	1.10	1.77	0.006	1.12	0.90	1.40	0.303
$40,000–60,000									1.26	0.97	1.63	0.080	1.04	0.82	1.33	0.729
$60,000–80,000									1.44	1.09	1.90	0.010	1.23	0.95	1.59	0.122
>$80,000									1.66	1.34	2.04	<0.001	1.24	1.02	1.50	0.034
Marital status																
Married									1.00				1.00			
Widowed									0.63	0.51	0.79	<0.001	0.81	0.67	1.00	0.047
Separated									0.96	0.78	1.18	0.704	1.06	0.87	1.28	0.580
Never married									0.58	0.49	0.69	<0.001	0.85	0.73	0.99	0.038
Smoking status																
Never									1.00				1.00			
Ever									1.14	0.98	1.33	0.098	1.10	0.95	1.27	0.207
Now									0.86	0.69	1.06	0.155	1.00	0.83	1.22	0.962
Insurance																
No	1.00				1.00				1.00				1.00			
Yes	1.08	0.32	3.59	0.902	0.81	0.25	2.68	0.732	1.30	1.14	1.49	<0.001	1.05	0.93	1.20	0.404
Smoke alarm installation																
Battery									1.00				1.00			
Hardwire									0.80	0.68	0.94	0.008	0.67	0.58	0.79	<0.001
Both									1.02	0.85	1.24	0.808	0.64	0.53	0.77	<0.001
Last tested smoke alarm																
Less than 6 months	1.00				1.00				1.00				1.00			
6 months or over	2.03	0.60	6.85	0.253	3.16	0.93	10.74	0.065	1.18	1.02	1.36	0.027	1.88	1.65	2.16	<0.001
Attitude towards fires																
Rating household related fire risk																
Low-risk	1.00				1.00											
High-risk	0.70	0.21	2.34	0.565	0.58	0.17	1.91	0.367								
Fire awareness																
Time have before whole room is on fire																
<5 min	1.00				1.00											
5–15 min	1.30	0.33	5.18	0.712	0.81	0.20	3.27	0.772								
Experienced unintentional or accidental fire																
Yes	1.00				1.00											
No	0.74	0.09	6.21	0.778	0.60	0.07	4.98	0.637								
Reflection about how you feel about fire in home																
High thought risk	1.00				1.00											
Low thought risk	0.59	0.17	2.10	0.415	1.48	0.43	5.15	0.536								
Practice home escape plan																
<1 year	1.00				1.00											
1 year or more	3.70	1.05	13.05	0.042	54.00	10.23	285.10	<0.001								
Living condition																
Own	1.00				1.00											
Rent	1.34	0.28	6.53	0.714	1.84	0.39	8.81	0.443								
Remoteness Index of Australia																
Highly accessible									1.00				1.00			
Accessible									0.93	0.73	1.19	0.566	0.91	0.72	1.14	0.395
Moderately accessible									0.85	0.68	1.06	0.159	0.77	0.62	0.95	0.015
Remote/Very remote									0.85	0.69	1.04	0.107	0.84	0.70	1.02	0.076

**Table 4:** Factors associated with home fire escape plan.

Variables	High-Risk Individuals								New South Wales (NSW) Population							
	Not Written				No Home Escape Plan				Not Written				No Home Escape Plan			
	OR	95% CI		*p*-Value	OR	95% CI		*p*-Value	OR	95% CI		*p*-Value	OR	95 %CI		*p*-Value
Language spoken at home																
English	1.00				1.00				1.00				1.00			
English plus other languages	0.24	0.07	0.89	0.033	0.50	0.12	2.01	0.329	0.44	0.34	0.56	<0.001	0.62	0.50	0.77	<0.001
Gender																
Male									1.00				1.00			
Female									1.36	1.14	1.62	0.001	0.99	0.84	1.16	0.866
Marital status																
Married									1.00				1.00			
Widowed									0.68	0.50	0.91	0.010	0.78	0.59	1.03	0.084
Separated									0.96	0.74	1.24	0.736	1.02	0.80	1.31	0.857
Never married									0.56	0.45	0.70	<0.001	0.74	0.60	0.90	0.003
Household income (before tax)																
<$20,000									1.00				1.00			
$20,000–40,000									1.29	0.99	1.69	0.058	1.12	0.87	1.43	0.383
$40,000–60,000									1.16	0.86	1.57	0.326	1.09	0.83	1.45	0.530
$60,000–80,000									1.38	0.99	1.93	0.056	1.28	0.93	1.74	0.125
>$80,000									1.46	1.12	1.89	0.005	1.30	1.02	1.66	0.036
Smoke alarm installation																
Battery									1.00				1.00			
Hardwire									0.71	0.58	0.88	0.001	0.60	0.49	0.73	<0.001
Both									0.87	0.68	1.10	0.252	0.57	0.45	0.71	<0.001
Last tested smoke alarm																
Less than 6 months									1.00				1.00			
6 months or over									1.27	1.05	1.55	0.016	1.90	1.58	2.28	<0.001
